# The Molecular Mechanism of PDE1 Regulation

**DOI:** 10.3390/cells14211722

**Published:** 2025-11-01

**Authors:** Jacob Nielsen, Morten Langgård, Josefine Fussing Tengberg, Jan Kehler

**Affiliations:** 1 Molecular and Single Cell Pharmacology, H. Lundbeck A/S, Valby, 2500 Copenhagen, Denmark; jotn@lundbeck.com; 2 Global Patents, H. Lundbeck A/S, Valby, 2500 Copenhagen, Denmark; mol@lundbeck.com; 3 Medicinal Chemistry III, H. Lundbeck A/S, Valby, 2500 Copenhagen, Denmark; jke@lundbeck.com

**Keywords:** PDE1, calcium activation, phosphodiesterase regulation, cAMP, cGMP

## Abstract

**Highlights:**

**What are the main findings?**

- The molecular mechanism of PDE1 calcium regulation is identified.
- PDE1 autoinhibition operates via a mechanism similar to that of PDE4.
- The activation mode of PDE1 is distinct and unique.

**What are the implications of the main findings?**

- A second mechanistic class of phosphodiesterase regulation is established.
- The findings may open new opportunities for pharmacological interventions targeting PDE1.

**Abstract:**

The phosphodiesterase 1 genes PDE1A, PDE1B, and PDE1C encode calcium-regulated cyclic nucleotide phosphodiesterases that mediate the interplay between calcium and cyclic nucleotide signaling in the brain, heart, and vasculature. While an inhibitory domain and a calmodulin-binding domain have been identified in PDE1, the mechanism of regulation is not understood. In this study, we investigated the regulatory mechanism through a series of experiments. The experimental data, supported by AlphaFold structure predictions, consistently point to the following model of PDE1 regulation: In the absence of calcium, the inhibitory domain of PDE1 binds to and blocks the catalytic site via molecular interactions that closely resemble those observed in autoinhibited PDE4. Upon calcium/calmodulin binding to PDE1’s calmodulin-binding domain, steric constraints prevent the inhibitory domain from reaching the catalytic site, thereby activating PDE1. Understanding this mode of PDE1 regulation may open new avenues for pharmacological intervention. Moreover, it establishes PDE1 and PDE4 as a second mechanistic class of phosphodiesterase regulation in addition to the GAF-domain-mediated regulation known to control the activity of several other PDEs.

## 1. Introduction

Phosphodiesterases (PDEs) are responsible for the degradation of the second messengers cyclic adenosine monophosphate (cAMP) and cyclic guanosine monophosphate (cGMP), thereby terminating cAMP signaling from G protein-coupled receptors and cGMP signaling from nitric oxide. There are 21 PDE genes, categorized into 11 families based on sequence similarity, inhibitor sensitivity, and other shared properties. PDEs differ in expression pattern, subcellular localization, cyclic nucleotide affinity (K_m_), turnover rate (V_max_), and regulation. These differences result in distinct biological effects of the PDEs and their inhibitors. For instance, PDE3 inhibitors are approved for cardiac failure, PDE4 inhibitors are approved for chronic obstructive pulmonary disease and psoriasis, and PDE5 inhibitors are approved for the treatment of erectile dysfunction [1].

The PDE1 family comprises three genes—PDE1A, PDE1B, and PDE1C—which encode dual substrate enzymes that degrade both cAMP and cGMP. PDE1A and PDE1B are highly expressed in the brain, where PDE1 activity accounts for a large proportion of the total cGMP degradation capacity [2]. PDE1A is also expressed in blood vessels and the heart, where it can influence vasodilation and heart rate increase [3,4,5]. The PDE1 inhibitor lenrispodun (ITI-214) is currently in clinical development for the treatment of Parkinson’s disease [6,7,8].

All PDEs share related catalytic domains in their C-terminal regions. In contrast, their N-terminal regions differ significantly and have in several cases been shown to control the activity of the catalytic domain. Little is known about the regulation of PDE families 3, 7, 8, and 9. The PDE families 2, 5, 6, 10, and 11 all contain two GAF domains in their N-terminal regions, which can bind either cAMP or cGMP. For PDE2A and PDE5, it has been clearly demonstrated that cGMP binding to their GAF domains activates the enzymes [9,10]. In PDE6, cGMP binding modulates interaction with its regulatory subunit [11,12], while data for cyclic nucleotide activation of PDE10A and PDE11A are more ambiguous [13,14]. A crystal structure of full-length PDE2A provided a breakthrough in the understanding of the GAF-domain-mediated regulation of PDE activity [15]. Like most other PDEs, PDE2A forms dimers, with the dimerization interface spanning the entire length of the protein. In the absence of cGMP, the substrate-binding pockets of the two subunits are in proximity, and the H-loops of the catalytic domains fold into the catalytic sites, thereby impeding catalytic activity. cGMP binding to GAF domain B leads to a conformational change of the dimer, which separates the catalytic domains, enabling enzymatic activation.

For PDE4, the longer isoforms that contain the N-terminal upstream conserved regions (UCR1 and UCR2) are activated by phosphorylation via the cAMP-activated protein kinase A (PKA), providing feedback regulation [16,17]. As for PDE2A, crystal structures of PDE4 that include both regulatory and catalytic domains have significantly advanced the mechanistic understanding of its regulation [18,19]. An α-helix from UCR2 was shown to bind to the entrance of the catalytic cleft, reducing V_max_ of the enzyme—a different mechanism than PDE2A, where inhibition is mediated by interaction between catalytic subunits. This PDE4 interaction is mediated by four key amino acids located on one side of the UCR2 α-helix [18]. PDE4 activation is controlled by PKA-mediated phosphorylation of UCR1, which is thought to cause UCR2 to move away from the catalytic cleft, although the precise molecular mechanism for this is unclear. The binding of the UCR2 α-helix over the catalytic cleft also explains the higher affinity of the PDE4 inhibitor rolipram for UCR2-containing isoforms compared to the catalytic domain alone, as rolipram interacts with both the catalytic cleft and the UCR2 α-helix [19]. This interaction further allows PDE4 isoform selectivity of some PDE4 inhibitors, due to sequence differences in the UCR2 α-helix [18].

PDE1s are unique among PDEs in being regulated by calcium/calmodulin, thus enabling crosstalk between calcium and cyclic nucleotide signaling, which are key signaling pathways in the brain and heart, where PDE1 expression is high [20]. As for PDEs 2 and 4, PDE1 regulation appears to involve de-repression: Deletion of the N-terminal part of PDE1A leads to constitutive activity comparable to that of activated full-length [21]. Sonnenburg et al. identified the N-terminal boundary of a putative inhibitory domain in PDE1A, and using peptides, they identified a calmodulin binding domain between the inhibitory and catalytic domains, which is critical for regulation [22]. However, the molecular mechanism by which calmodulin binding to PDE1 leads to activation has not been identified—is PDE1 regulated by a PDE2-like, a PDE4-like, or an unrelated mechanism?

## 2. Materials and Methods

### 2.1. Constructs and Alignments

Human PDE1B1 (Refseq: NM_000924.4) and its N-terminal deletion constructs were cloned into BamH1 and Xho1 sites of pcDNA3.1(+) using PCR. The stated boundary and amino acid positions for mutants and deletion constructs are based on the PDE1B1 isoform (Refseq: NP_000915). For all N-terminal constructs, a start codon was introduced upstream of the indicated boundary, resulting in a methionine preceding the amino acid specified by the N-terminal boundaries. Point mutations and internal deletions were generated using the QuickChange^TM^ site-directed mutagenesis method, as previously described [23]. The PDE1B-PDE4D chimera was generated by inserting the human PDE4D catalytic domain with a PCR-introduced N-terminal BspE1 site into a pcDNA3.1-PDE1B (82-end) construct using an internal BspE1 site at the junction of the regulatory and catalytic domain and an Xho1 site downstream of the stop codon. The amino acid sequence around the fusion point is shown in Figure S1. All constructs were validated through sequencing. Maps and sequences are available upon request. Alignments were generated using Geneious Prime (v 2024.0.7). Amino acids are colored according to the RasMol color scheme, and similarities are indicated according to the BLOSUM substitution matrix [24].

### 2.2. Phosphodiesterase Expression

Plasmids were transiently transfected into HEK293 cells using Lipofectamine 2000 (Thermo Fisher, Waltham, MA, USA) according to the manufacturer’s instructions. For each well of a 6-well plate, 5 µg plasmid and 10 µL Lipofectamine 2000 were used.

Next, 48 h after transfection, cells were washed once with ice-cold PBS, scraped off into 2 mL PBS, and pelleted via centrifugation at 700× *g* for 5 min at 4 °C. The resulting cell pellet was frozen. Later, the pellet was thawed and resuspended in 0.5 mL of lysis buffer 50 mM Tris-HCL (pH 8.0) (Sigma-Aldrich, T3253, St. Louis, MO, USA), 1 mM MgCl_2_, and Complete ULTRA protease inhibitor cocktail (Roche, 05892970001, Basel, Switzerland). The lysate was then centrifuged at 20.000× *g* for 10 min at 4 °C to remove nuclei and membranes. Then, 0.05% Tween20 (Sigma-Aldrich, P2287) (final concentration) was added to the supernatant containing the cytosolic PDE1 before it was aliquoted and snap frozen.

### 2.3. Phosphodiesterase Assays

PDE activity was measured using a scintillation proximity assay (SPA)-based method, similar to that previously described [13]. Briefly, assays were conducted in 96-well plates using a buffer containing 40 mM Hepes (pH 7.2), 140 mM KCl, and 1.5 mM MgCl_2_. In each well, 0.005–0.05 µL PDE lysate was mixed with or without final concentrations of 50 µM CaCl_2_ and 50 nM calmodulin (Sigma-Aldrich, P1431) in a volume of 55 µL, and allowed to stand for 5 min at room temperature. Lysate amounts were adjusted to ensure that less than 30% of the tritiated substrate was hydrolyzed. Next, 10 µL tritiated cyclic nucleotide substrate ([3H]-cGMP for PDE1 and [3H]-cAMP for PDE4 and the PDE1/4 chimera) was added, and the reaction was allowed to proceed for 1 h at room temperature before 15 µL of 8 mg/mL phosphodiesterase SPA beads (Perkin Elmer, RPNQ0150, Shelton, CT, USA) was added. After another hour at room temperature to allow binding of the reaction products to the beads, the plates were counted for 2 min using a liquid scintillation counter (Wallac Trilux, Turku, Finland). For PEP1-sol, a slight absorbance-driven reduction in signal was observed at high concentrations (≈25% at 120 µM). To avoid overestimating inhibition, PEP1-sol was also tested by adding it after reaction termination (i.e., after SPA bead addition) in concentration–response experiments, and pre-addition inhibition minus post-addition (apparent) inhibition at each concentration was used for IC_50_ calculations. Since absorbance was low, this had only a minor impact on IC_50_ estimates. Peptides were synthesized at Genscript at >98% purity.

### 2.4. Structure Predictions

The structure of the inhibitory domain peptide (SDAVPSEVRDWLASTFTQQ) was predicted using PEP-FOLD3 [25]. PyMOL (v3.1.3, Schrödinger, LLC) was used for rendering. The structure of PDE1B1 (Refseq: NM_000924.4) and truncated PDE1B without the terminal regions that are dispensable for activation (amino acids 82–501 of Refseq: NM_000924.4) were predicted using AlphaFold-Multimer AF2.3 [26,27,28]. The top-ranked structure predictions were visualized using MOE (v2024.6, Chemical Computing Group Inc.).

### 2.5. Statistics and Data Analysis

Statistical analysis and curve fits were performed using GraphPad Prism (v10.1.2). All statistical evaluations were based on central estimates from at least three independent experiments.

## 3. Results

### 3.1. PDE1B Activation and Regulatory Domain Boundaries

**Figure 1:**
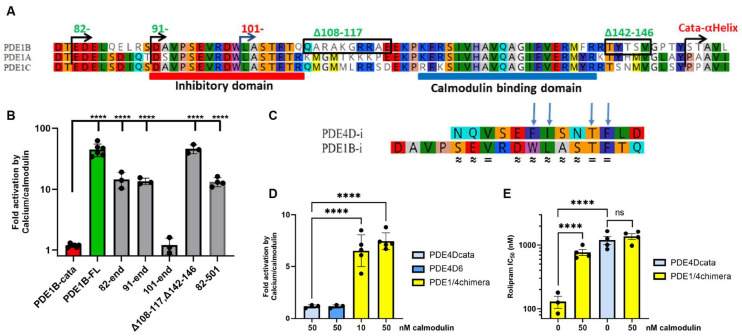
The PDE1 inhibitory domain has functional similarity to the PDE4 inhibitory domain. (**A**) Alignment of regulatory region from human PDE1A, PDE1B, and PDE1C. Boundaries of PDE1B deletion constructs and start of the first α-helix in the catalytic domain X-ray [29] are indicated above the alignment. N-terminal boundaries of constructs that retain calcium/calmodulin regulation are shown in green. Proposed inhibitory and calmodulin-binding domains are marked below the alignment. (**B**) Activation of full-length PDE1B (PDE1B-FL; 1–536) and PDE1B deletion constructs by 50 nM calmodulin and 50 µM CaCl_2_, compared to the PDE1B catalytic domain (PDE1B-cata; 146–536). Numbers indicate boundaries of deletion constructs based on PDE1B1 (NP_000915)—see Figure S5 for the full sequence. (**C**) Alignment of the PDE1B inhibitory domain (PDE1B-i) with the inhibitory α-helix from PDE4D (PDE4D-i). The four amino acids critical for PDE4D-i interaction with the PDE4D catalytic domain [18] are marked with arrows. Amino acid identities (=) and similarities (≈) are indicated below the alignment. (**D**) A PDE1/4 chimera with the PDE1B regulatory domain fused to the PDE4D catalytic domain is regulated by calcium/calmodulin, while the PDE4D catalytic domain (PDE4Dcata) and PDE4D6 are not. 50 µM CaCl_2_ was added along with the indicated concentrations of calmodulin. (**E**) Rolipram IC_50_ values. Rolipram inhibits the PDE1/4 chimera more potently than the PDE4D catalytic domain alone. This increased potency is lost upon activation of the chimera by calcium/calmodulin. All values are presented as means ± SEM from 3–5 independent experiments. Statistical significance was determined on log-transformed data using one-way ANOVA with Dunnett’s (**B**) and (**D**) or Sidak’s (**E**) multiple comparisons correction. Ns: nonsignificant, and **** *p* < 0.0001.

To confirm previously published PDE1 activation data, we expressed full-length PDE1B and the PDE1B catalytic domain in HEK293 cells and measured PDE activity in the presence or absence of Ca^2+^ and calmodulin. As expected, we observed a dramatic activation of full-length PDE1B (PDE1B-FL), but not the PDE1B catalytic domain (PDE1B-cata), upon calcium/calmodulin stimulation (Figure 1A,B). Testing of N-terminal deletion constructs (Figure 1B) supported the previously reported N-terminal boundary required for regulatory function [22]. We also examined the effect of deleting 35 amino acids from the C-terminal to the catalytic domain (PDE1B_82–501) and found no impact on calmodulin regulation of activity, indicating that regulation is mediated exclusively by the N-terminal domain (Figure 1B). Further, we tested a PDE1B construct with internal deletions between the proposed inhibitory, calmodulin-binding, and catalytic domains (D108–117, D142–146—see Figure 1A). This construct also retained calcium/calmodulin regulation of activity, supporting the proposed boundaries for the inhibitory and calmodulin-binding domains and demonstrating that the regions between them are not required for regulation (Figure 1A,B). Consistent with this, conservation among PDE1A, PDE1B, and PDE1C is close to 100% in the proposed inhibitory and calmodulin-binding domains, but low in the connecting regions (Figure 1A and Figure S2).

### 3.2. The PDE1B Inhibitory Domain Has Similarities to PDE4 and Can Regulate PDE4 Activity in a PDE1/4 Chimera

Blast and similar searches did not reveal any obvious overall sequence similarity between PDE1s and other PDE families outside the catalytic domain. However, when we specifically compared the inhibitory domain of PDE1B to the 13-amino acid inhibitory α-helix of PDE4D [18], we observed three amino acid identities and seven amino acid similarities (Figure 1C). Notably, all four amino acids in the PDE4D inhibitory domain that interact with its catalytic domain [18] were identical or similar in the PDE1B inhibitory domain (Figure 1C). Furthermore, the separation between the inhibitory and catalytic domains is comparable in PDE1B and PDE4D: In PDE1B, there are 46 amino acids between the conserved phenylalanine in the inhibitory domain and the start of the first α-helix in the catalytic domain, compared to 42 amino acids in PDE4D (Figure S1).

This similarity suggests a similar molecular mechanism for PDE1 and PDE4 inhibitory domains. To test whether the regulatory domains are functionally transferable, we generated and expressed a PDE1/4 chimera with the regulatory domain from PDE1B fused to the catalytic domain from PDE4D (Figure S1). This construct exhibited clear calcium/calmodulin-regulated PDE4 activity (Figure 1D). As expected, the chimera’s activity could be inhibited with selective PDE4 inhibitors such as rolipram (Figure 1E) and roflumilast (Figure S1B). Rolipram is a selective PDE4 inhibitor with higher potency towards PDE4 isoforms containing regulatory domains, due to its interaction with both the inhibitory and catalytic domains [19]. Strikingly, rolipram inhibited the PDE1/4 chimera more potently than the PDE4D catalytic domain (Figure 1E), suggesting that the PDE1B inhibitory domain in the PDE1/4 chimera can interact with rolipram in the catalytic site of PDE4D similarly to its published interaction with the PDE4D inhibitory domain [18]. Moreover, rolipram affinity boost was lost when the chimera was activated by calcium/calmodulin (Figure 1E), indicating that calcium/calmodulin binding moves the inhibitory domain away from the catalytic cleft, leading to loss of that interaction.

### 3.3. Alanine Scan of the PDE1B Inhibitory Domain

**Figure 2:**
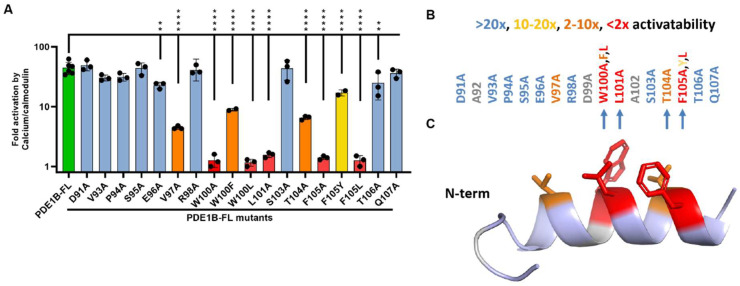
Alanine scan of the PDE1B inhibitory domain identifies key amino acids. (**A**) Activation by 50 nM calmodulin and 50 µM CaCl_2_ of full-length PDE1B (PDE1B-FL) with mutations of individual amino acids from the inhibitory domain compared to wild-type PDE1B-FL. Values are presented as means ± SEM from 3–5 independent experiments; significance was determined using one-way ANOVA with Dunnett’s multiple comparisons correction on log-transformed data. ** *p* < 0.01, and **** *p* < 0.0001. (**B**) Overview of the impact of inhibitory domain mutations on the calcium/calmodulin activation potential of PDE1B. The four amino acids that align with the amino acids required for PDE4 regulation are marked with arrows. (**C**) PEP-FOLD3 [25] predicted structure of the PDE1B inhibitory domain with the five key amino acids highlighted. Unmutated PDE1B-FL is shown in green. Substitutions that abolished activation (<2× activatability) in red, large effect substitutions (2–10× activatability) in orange, medium (10–20×) activatability in yellow, and minimal-no effect (>20× activatability) mutants in blue. Mutations of amino acids in grey were not tested (A92 and A102 were already alanines, and there was too low expression of D99A).

To further investigate the PDE1B inhibitory domain, we performed an alanine scan of the inhibitory domain within the context of full-length PDE1B and assessed the impact on activation by calcium/calmodulin (Figure 2). Most alanine substitutions did not affect activation, but three mutations (W100A, L101A, and F105A) essentially abolished calcium/calmodulin activation, while two others (V97A and T104A) substantially reduced it. These five residues are very strongly conserved in PDE1s across animal kingdoms and in humans (Figure S2). Strikingly, four of these five positions (W100, L101, T104, and F105) align with the four amino acids shown to interact with the catalytic domain in the PDE4D crystal structure (Figure 2B). We also tested more conservative substitutions of the key aromatic residues W100 and F105. Aromatic substitutions (W100F and F105Y) were tolerated, albeit with some reduction in PDE1B activatability, whereas lipophilic substitutions to leucine were not tolerated (Figure 2A). We assessed the likely secondary structure of the PDE1B inhibitory domain using PEP-FOLD [30], which predicted that most of the domain adopts an α-helical conformation (Figure 2C), similar to the inhibitory domain of PDE4D [18]. Notably, the five residues identified as critical for calcium/calmodulin activation are all predicted to be on the same side of the α-helix, consistent with a role in interaction with the catalytic domain.

### 3.4. Inhibitory Domain Peptides Can Inhibit PDE1B in Trans

**Figure 3:**
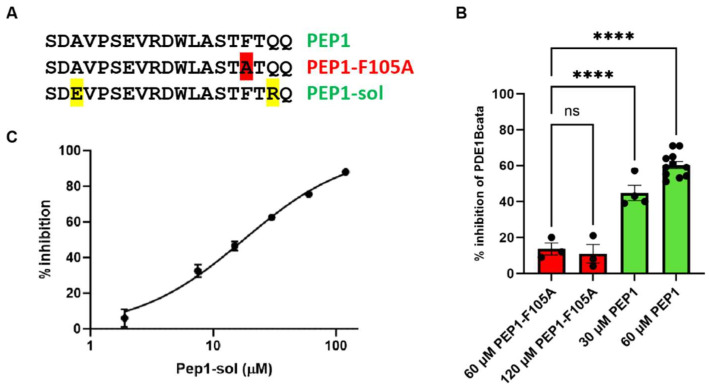
Inhibition of the PDE1B catalytic domain by the inhibitory domain peptide added in trans. (**A**) Sequence of PEP1 encompassing the proposed PDE1B inhibitory domain, PEP1-F105A with mutation of a phenylalanine (highlighted in red) required for inhibitory control in full-length PDE1B, and PEP1-sol with solubility-enhancing mutations of two amino acids that are not phylogenetically conserved (highlighted in yellow). Mutations are highlighted. (**B**) % inhibition of PDE1B catalytic domain (PDE1B-cata) enzyme activity by PEP1 (green) compared to 60 µM PEP1-F105A (red). Significance was determined using one-way ANOVA with Dunnett’s multiple comparisons correction on log-transformed data; ns, nonsignificant and **** *p* < 0.0001. (**C**) Representative PEP1-sol concentration–response of PDE1B-cata inhibition. Average IC_50_ ± SEM from 3 independent experiments was 17.9 ± 2.1 µM.

In a PDE4-like regulation mechanism, the inhibitory region might bind and inhibit the PDE1B catalytic domain when added in trans, though presumably with low affinity, as it would be an intermolecular rather than intramolecular interaction. Proper peptide folding is, of course, required; however, relevant α-helix folding is supported by PEP-FOLD predictions (Figure 2C). To test this, we synthesized a peptide (PEP1) encompassing the proposed inhibitory domain and assessed its effect on the catalytic activity of PDE1B-cata (Figure 3). For comparison, we tested a variant of the peptide (PEP1-F105A), in which the phenylalanine—identified in our alanine scan as essential for inhibitory domain function (Figure 2)—was mutated to alanine. As predicted, PEP1 concentration-dependently inhibited PDE1B-cata activity, whereas PEP1-F105A did not (Figure 3B). Due to solubility limitations, PEP1 did not permit full concentration–response curves. To address this, we designed a more soluble variant (PEP1-sol, Figure 3A) by mutating two amino acids of PEP1 to charged amino acids. To minimize the risk of a functional impact of the mutations, positions without phylogenetic conservation near the ends of PEP1 were selected, and the residues were mutated to amino acids that were present in PDE1s from other species (Figure S2). As predicted, PEP1-sol also inhibited PDE1B-cata activity and enabled complete concentration–response, where it inhibited PDE1B-cata activity with an IC_50_ of 17.9 ± 2.1 μM (Figure 3C), which is compatible with the inhibition observed with the original PEP1 peptide (Figure 3B).

### 3.5. Calmodulin EC_50_ Inversely Correlates with Maximal Activation of Inhibitory Domain Mutants

**Figure 4:**
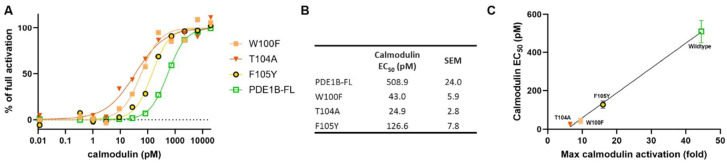
Less calmodulin is needed for activation of PDE1B mutants with reduced activatability. The impact of calmodulin on the activation of full-length PDE1B (PDE1B-FL) and PDE1B-FL with individual mutations that reduce activatability was examined in calmodulin concentration–response experiments in the presence of 50 µM CaCl_2_. (**A**) Representative example of calmodulin concentration–response’s impact on activation of the different constructs. (**B**) Table with average calmodulin activation EC_50_ and SEM derived from 5 independent experiments. (**C**) Correlation between maximal calmodulin activation and calmodulin EC_50_ for the different PDE1B mutants. Simple linear regression coefficient of determination, R^2^ = 0.98.

A simple mechanism for activation of PDE1 by calcium/calmodulin could be that calmodulin binding between the inhibitory and catalytic domains sterically hinders the inhibitory domain from reaching the catalytic cleft. If this is the case, calmodulin binding to the calmodulin binding domain would indirectly compete with the inhibitory domain binding to the catalytic cleft. Consequently, inhibitory mutants with reduced affinity for the catalytic cleft would be predicted to require less calmodulin to shift the equilibrium, resulting in lower calmodulin EC_50_. To test this, we selected inhibitory domain mutants that exhibited reduced activatability, presumably reflecting lower affinity for the catalytic cleft. Calmodulin concentration–response experiments confirmed that these indeed have lower calmodulin EC_50_ values and that calmodulin EC_50_ inversely correlates with their maximal activation (Figure 4).

### 3.6. Structure and Model

**Figure 5:**
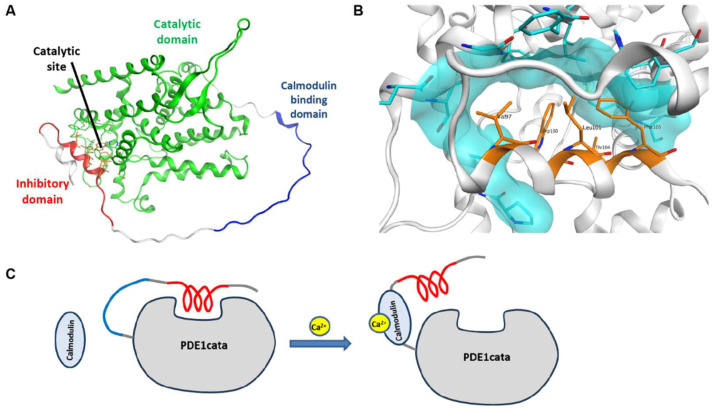
AlphaFold structure and model for activation. (**A**) AlphaFold structure prediction of PDE1B residues 82–501. The catalytic domain is colored green, the calmodulin binding domain is colored blue, and the inhibitory domain is colored red. (**B**) Blowup of the predicted binding of the inhibitory domain α-helix to the catalytic site of the catalytic domain. The five key residues of the inhibitory domain that were identified in the alanine scan are labelled and highlighted in orange. They are predicted to contact several residues around the catalytic site, the surfaces of which are highlighted in cyan. (**C**) Activation model of PDE1. In the absence of calcium, the inhibitory domain (red) binds to and blocks the catalytic site. When cytoplasmic calcium levels are increased, the calcium/calmodulin complex binds to the PDE1 calmodulin-binding domain, which sterically hinders the inhibitory domain from reaching the catalytic site, thus leading to activation of PDE1.

Despite several attempts, we were unable to crystallize full-length PDE1B or truncated constructs that include the inhibitory domain. Instead, we tested if AlphaFold could generate a structural model consistent with our experimental observations. To focus on the functionally relevant regions, we used PDE1B without the terminal regions that are dispensable for activatability (amino acids 82–501) for structure prediction. Strikingly, AlphaFold predicted that the inhibitory domain folds into an α-helix that binds into the catalytic cleft (Figure 5A,B), in agreement with our experimental findings. Moreover, the five inhibitory domain residues identified as critical for PDE1B regulation in the alanine scan (Figure 2) were predicted to interact directly with the catalytic cleft (Figure 5B). In addition to blocking access to the catalytic site, the key inhibitory domain residues are predicted to insert deeply into the catalytic cleft, which likely precludes binding of cyclic nucleotides and inhibitors. The calmodulin binding domain and other residues between the inhibitory and catalytic domains were predicted to be largely unstructured, although the confidence of the structure prediction for that region is low (Figure S4). We also predicted the structure of the full-length PDE1B1 and obtained similar results (Figure S4). Together, the experimental data and AlphaFold predictions support the activation model shown in Figure 5C.

## 4. Discussion

All data generated support the following model of PDE1 regulation: In the absence of calcium/calmodulin activation, the PDE1 inhibitory domain binds to and blocks access to the catalytic cleft, with a similar binding mode as that previously reported for PDE4 (Figure 5C). Upon binding of calcium-activated calmodulin to the calmodulin-binding domain, this interaction is abolished, allowing PDE1 to become active.

This model is supported by several lines of evidence: The similarity between the PDE1 inhibitory domain and the PDE4 inhibitory α-helix; key amino acids identified in the alanine scan; the transferability of regulation to PDE4 with enhancement of rolipram binding; AlphaFold predictions; and inhibition by an inhibitory peptide in trans—all of which indicate binding of the PDE1 inhibitory domain to the catalytic cleft in the absence of calcium/calmodulin in a manner similar to what was described for PDE4. When activated or disinhibited, the enhancement of rolipram potency of the PDE1/4 chimera is lost, suggesting that the inhibitory domain is no longer close to the catalytic site. Since the calmodulin-binding domain constitutes around half of the residues between the inhibitory and catalytic domains, it is plausible that calmodulin binding imposes structural constraints that prevent the inhibitory domain from reaching the catalytic cleft. Furthermore, it is supported by the lower calmodulin EC_50_ values observed in mutants with inhibitory domain mutations that reduce regulation: If inhibitory domain binding to the catalytic cleft excludes and thus competes with calmodulin binding to the calmodulin-binding domain, then weaker binding of the inhibitory domain would require less calmodulin to outcompete it.

The AlphaFold prediction of the PDE1B structure aligns well with the experimental data, as it predicts the PDE1B inhibitory α-helix to bind and occlude the catalytic cleft in a PDE4-like binding mode. The region connecting the inhibitory and catalytic domains is predicted to be a largely unstructured loop, linking the C-terminus of the inhibitory domain to the first α-helix of the catalytic domain, located on the opposite side of the catalytic domain. As expected for an unstructured region, the model confidence for this intervening region is low, and phylogenetic conservation of individual residues within this intervening region is low, except for the calmodulin binding site. However, the approximate length of the region is conserved across species and subtypes, consistent with its proposed role as a flexible spacer. The considerable distance spanned by this spacer supports the hypothesis that constraints and steric restrictions of calmodulin binding prevent the inhibitory domain from reaching the catalytic cleft, leading to disinhibition. While the AlphaFold models represent a monomer, PDE1 is known to form dimers. The AlphaFold model is compatible with inhibition and activation within each subunit, but cross-inhibition between subunits—as observed in PDE4—cannot be excluded based on current data.

We observed a calcium/calmodulin activation potential of approximately 40-fold for PDE1B1, whereas Sonnenburg et al. observed around 7-fold activation of PDE1A1 [22]. One potential explanation for this discrepancy is the different origins of the enzymes: we used untagged human PDE1B expressed in mammalian cells, while Sonnenburg et al. used bovine PDE1A constructs with N-terminal HIS-tags expressed in insect cells. In mouse cortical lysate, we have observed PDE1 calcium/calmodulin activation potential of more than 40-fold (Figure S3). Assay buffer and other experimental conditions may also contribute to the observed differences. Additionally, proteolytic cleavage of a proportion of PDE1 could result in a population of uninhibited PDE1, thereby reducing the apparent activation potential. In contrast, there is no obvious mechanism by which the activation potential could be artificially increased. Thus, the calcium/calmodulin control of PDE1 activity is probably even tighter than previously appreciated, potentially representing the tightest regulation reported for any PDE. Presumably, only a small proportion of PDE1 is activated at any given timepoint in the brain, since cytosolic calcium concentration is low in most neurons at any given timepoint.

While the inhibitory domains of PDE1 and PDE4 appear to function through similar molecular interactions with their respective catalytic clefts, the regulation of these interactions and thus the activation mechanisms are very different. Obviously, PDE1 is regulated by calcium-controlled calmodulin binding, while PDE4 is regulated by cAMP-controlled PKA phosphorylation. Further, the calmodulin-binding domain in PDE1 is located between the inhibitory and catalytic domains, allowing disinhibition to occur through simple steric constraints upon calcium-activated calmodulin binding. In contrast, the PDE4 PKA phosphorylation site that mediates activation is located N-terminal to the inhibitory domain within UCR1, and phosphorylation can disrupt UCR1-UCR2 interaction and dimerization [31,32]. Unlike for PDE4, PDE1 dimerization does not depend on its regulatory domain [22].

In summary, we have identified the mechanism by which PDE1 is activated by calcium/calmodulin. This mechanism is related to that of PDE4 in that both involve similar inhibitory domains capable of binding to and blocking the catalytic site. However, the mechanisms of activation differ between the two. Together, PDE1 and PDE4 represent a second mechanistic class of PDE regulation, distinct from the GAF domain-regulated PDEs. Further research may reveal whether additional PDEs are regulated through similar mechanisms, and whether new types of PDE1 inhibitors that contact both the inhibitory and catalytic domains are allowed, as in the new generation of PDE4 inhibitors.

## Data Availability

Data is contained within the article or Supplementary Material.

## Supplementary Materials

The following supporting information can be downloaded at https://www.mdpi.com/article/10.3390/cells14211722/s1. Figure S1: PDE1/4 chimera sequence comparison and inhibition by roflumilast. Figure S2: Conservation of the PDE1B regulatory region. Figure S3: PDE1 activation in mouse cortical lysate. Figure S4: Additional AlphaFold poses of PDE1B. Figure S5: PDE1B sequence and domains.
