# Supplementary material for: The Molecular Mechanism of PDE1 Regulation
Source: Cells. 2025 Nov 1;14(21):1722. doi: 10.3390/cells14211722 (PMC12610503; doi:10.3390/cells14211722)
Supplement: Supplementary file 1 [file cells-14-01722-s001.zip › cells-3935491-supplementary.pdf]

Supplementary Materials

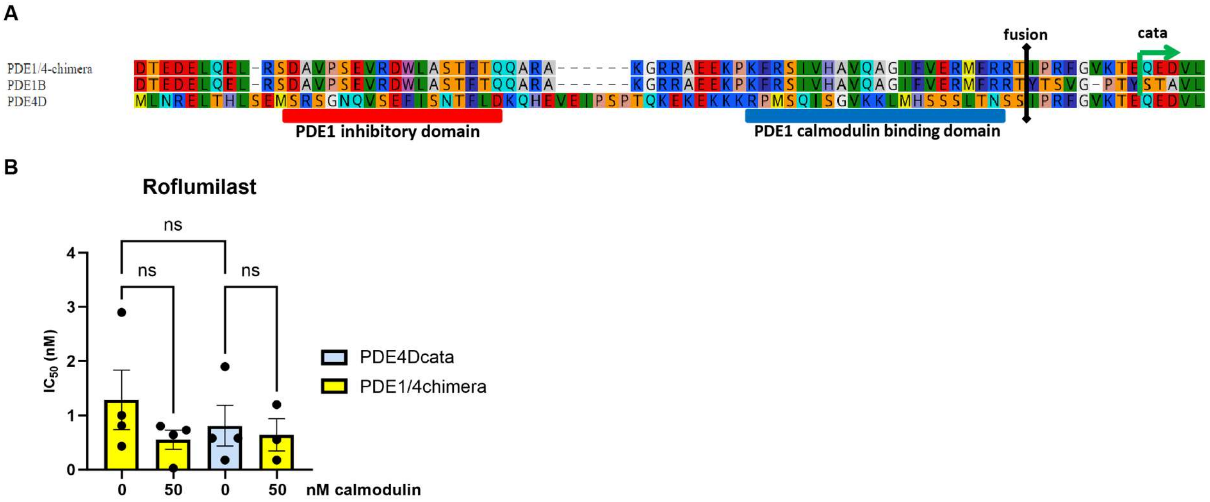

Figure S1: PDE1/4 chimera sequence comparison and inhibition by roflumilast. (A) Alignment of the core regulatory region of the PDE1/4 chimera compared to PDE1B and PDE4D. N-terminal to the point of fusion (marked), the chimera is identical to PDE1B, C-terminal to that, it is identical to PDE4D. **(B)** The selective PDE4 inhibitor roflumilast inhibits the PDE1/4 chimera. IC_{50} towards the chimera is not significantly different from PDE4D catalytic domain (PDE4Dcata) and is unaffected by calcium/calmodulin activation. All values are presented as means ± SEM from 3-4 independent experiments; significance was determined using one-way ANOVA with Sidak's multiple comparisons correction on log-transformed data.

A

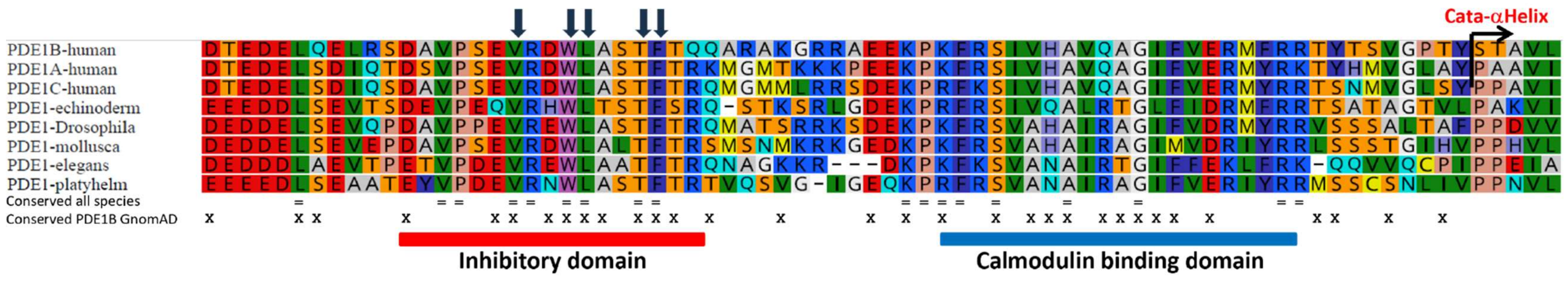

Figure S2: Conservation of PDE1B regulatory region. Alignment of the regulatory regions from the three human PDE1 isoforms and paralogues from phylogenetically distant species. Arrows mark the five amino acids in the inhibitory domain that were identified to be important for regulatory function in the alanine scan (Figure 2) – all five are phylogenetically conserved. Below the alignment, positions that are identical in all species are marked with “=“. Below that, positions that have no registered human PDE1B mutations in gnomAD are marked with “X” [1]. Below that, the proposed inhibitory and calmodulin-binding domains are marked. PDE1-echinoderm (Strongylocentrotus purpuratus, NP_001091918), PDE1-Drosophila (Drosophila melanogaster, NP_001245994), PDE1-mollusca (Gigantopelta aegis, XP_041373981), PDE1-elegans (Caenorhabditis elegans, NP_001129790), PDE1-platyhelm (Schistosoma haematobium, XP_035588137).

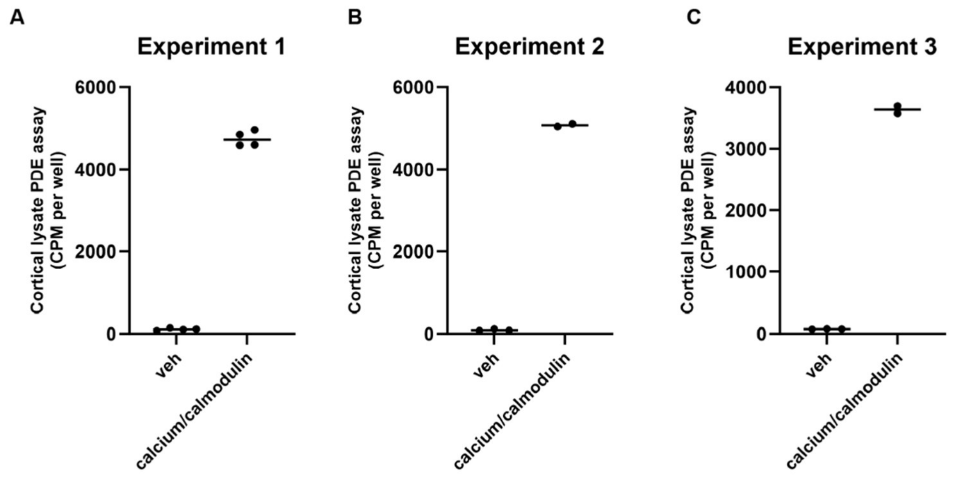

Figure S3: PDE1 activation in mouse cortical lysate. Mouse cortex was lysed as described for cells in materials and methods. [3H]-cGMP PDE activity was measured as described in materials and methods except that 50 nM of the selective PDE10A inhibitor MP10 and 50 nM of a selective PDE2A inhibitor were added to remove contribution from those phosphodiesterases. 0.003-0.01 µl cortical lysate was added per 96-well plate well. **(A-C)** Graphs show background subtracted signal observed in three independent experiments in the absence or presence of 50 µM $CaCl_2$ and 50 nM calmodulin. Dots in each graph represent technical replicates. 42-, 52- and 47-fold increase in PDE activity by calcium/calmodulin were observed in experiments 1-3, respectively.

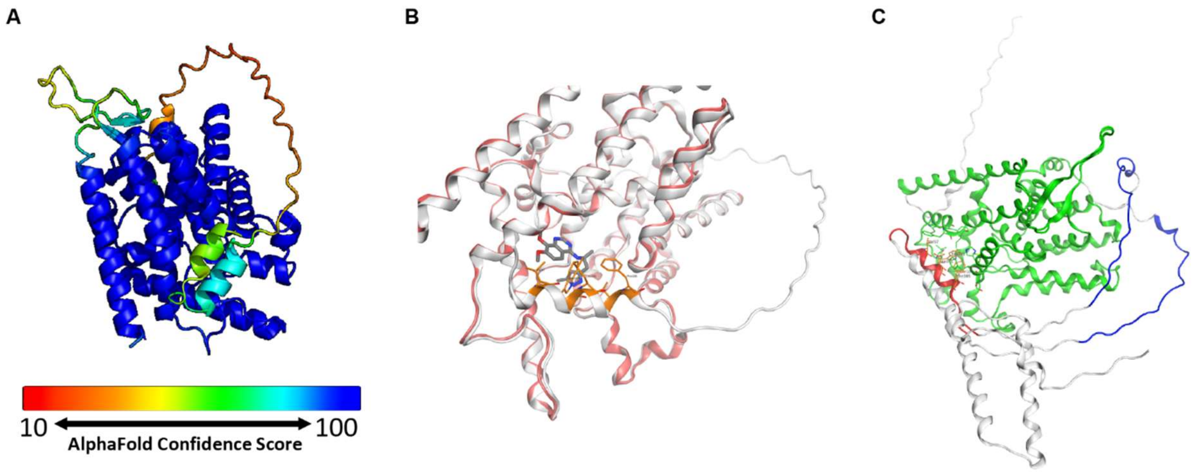

Figure S4: Additional AlphaFold poses of PDE1B. (A) AlphaFold structure prediction of PDE1B residues 82-501 (as in Figure 5A) colored according to AlphaFold confidence score. Confidence scores are high for catalytic domain, medium-good for inhibitory domain, and low for unstructured regions. **(B)** AlphaFold (white) overlayed with PDB 5W6E catalytic domain Xray structure in pink [2]. The AlphaFold and Xray structures have close to perfect overlap. The inhibitor from that structure (compound (S)-3) is also shown - it clashes with amino acids from the inhibitory domain, suggesting that there would not be room for it in the inactive form of the catalytic domain. **(C)** AlphaFold structure prediction of full-length PDE1B1 (Refseq NM_000924.4). The catalytic domain is colored green, calmodulin binding domain is colored blue, and the inhibitory domain is colored red as in Figure 5A. The structure prediction of the overlapping residues is very similar to that of the truncated construct (82-501) shown in Figure 5A.

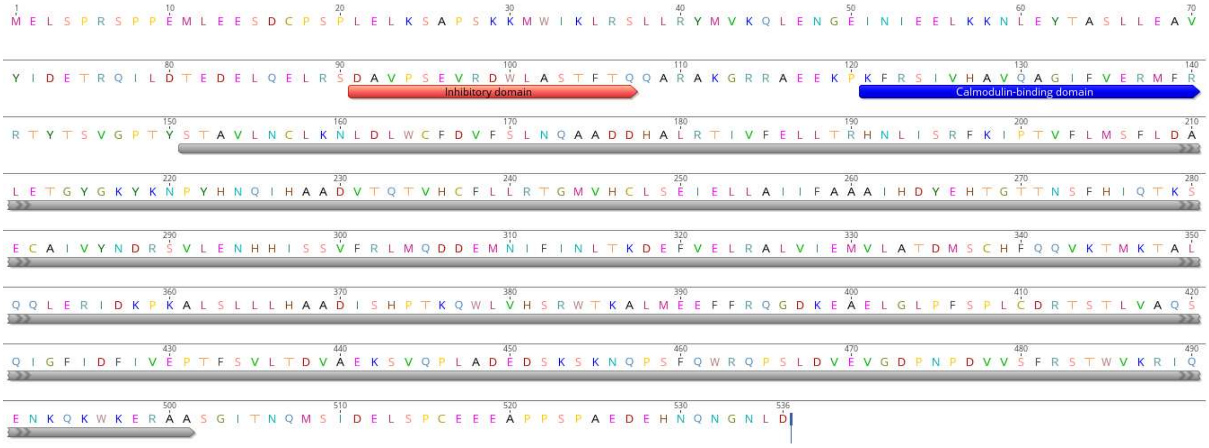

Figure S5: PDE1B sequence and domains. PDE1B inhibitory and calmodulin-binding domains as defined in the paper are marked underneath with red and blue, respectively. The catalytic domain defined by the first to the last α-helix in the published PDE1B catalytic domain PDE1B Xray structure [2] are marked with a grey bar.

References

1. Chen, S., L. C. Francioli, J. K. Goodrich, R. L. Collins, M. Kanai, Q. Wang, J. Alfoldi, N. A. Watts, C. Vittal, L. D. Gauthier, T. Poterba, M. W. Wilson, Y. Tarasova, W. Phu, R. Grant, M. T. Yohannes, Z. Koenig, Y. Farjoun, E. Banks, S. Donnelly, S. Gabriel, N. Gupta, S. Ferriera, C. Tolonen, S. Novod, L. Bergelson, D. Roazen, V. Ruano-Rubio, M. Covarrubias, C. Llanwarne, N. Petrillo, G. Wade, T. Jeandet, R. Munshi, K. Tibbetts, Consortium Genome Aggregation Database, A. O'Donnell-Luria, M. Solomonson, C. Seed, A. R. Martin, M. E. Talkowski, H. L. Rehm, M. J. Daly, G. Tiao, B. M. Neale, D. G. MacArthur, and K. J. Karczewski. "A Genomic Mutational Constraint Map Using Variation in 76,156 Human Genomes." *Nature* 625, no. 7993 (2024): 92-100.
2. Humphrey, J. M., M. Movsesian, C. W. Am Ende, S. L. Becker, T. A. Chappie, S. Jenkinson, J. L. Liras, S. Liras, C. Orozco, J. Pandit, F. F. Vajdos, F. Vandeput, E. Yang, and F. S. Menniti. "Discovery of Potent and Selective Periphery-Restricted Quinazoline Inhibitors of the Cyclic Nucleotide Phosphodiesterase Pde1." *J Med Chem* 61, no. 10 (2018): 4635-40.
